# Remission of HPV-Related Diseases by Antivirals for Herpesvirus: Clinical Cases and a Literature Review

**DOI:** 10.3390/v16050756

**Published:** 2024-05-10

**Authors:** Maria Balestrieri, Maria Vincenza Chiantore, Anna Rosa Garbuglia, Caterina Carnovale-Scalzo, Susanna Falcucci, Paola Di Bonito

**Affiliations:** 1 Gynaecology and Diagnostic Colposcopy Clinic, Via Enea, 23, 00181 Rome, Italy; dmbalestrieri@gmail.com; 2 Department of Infectious Diseases, Viral Hepatitis and Oncovirus and Retrovirus Diseases (EVOR) Unit, Istituto Superiore di Sanità, Viale Regina Elena 299, 00161 Rome, Italy; mariavincenza.chiantore@iss.it (M.V.C.); susanna.falcucci@iss.it (S.F.); 3 Laboratory of Virology, National Institute for Infectious Diseases (INMI) Lazzaro Spallanzani, IRCCS, Via Portuense, 292, 00149 Rome, Italy; annarosa.garbuglia@inmi.it; 4 Histopathology Laboratory, Ospedale San Carlo di Nancy, GVM Care and Research, Via Aurelia, 275, 00165 Rome, Italy; kytos.sd@gmail.com

**Keywords:** human papillomavirus, warts, condyloma, acyclovir and valaciclovir

## Abstract

Epidemiological studies have shown that HPV-related diseases are the most prevalent sexually transmitted infections. In this context, this report will present various clinical cases demonstrating the effectiveness of Acyclovir (ACV) or its prodrug Valaciclovir (VCV), both acyclic guanosine analogs commonly used for the treatment of HHV-1 and HHV-2, for the treatment of HPV-related diseases. The report shows the remission of five cases of penile condyloma and a case of remission in a woman affected by cervical and vaginal condylomas and a vulvar giant condyloma acuminate of Buschke and Lowenstein. The literature review shows that ACV is effective in treating skin warts when administered orally, topically, and intralesionally, suggesting its therapeutic potential in other diseases associated with HPV. ACV was also used successfully as an adjuvant therapy for juvenile and adult forms of laryngeal papillomatosis, also known as recurrent respiratory papillomatosis, prolonging the patient’s symptom-free periods. Although the prevention of HPV infections is certainly achieved with the HPV vaccine, ACV and VCV have shown to be effective even against genotypes not included in the current vaccine and can be helpful for those problematic clinical cases involving unvaccinated individuals, immunocompromised patients, people who live with HIV, or non-responders to the vaccine. We and others concluded that randomized clinical trials are necessary to determine the efficacy of ACV and VCV for HPV-related diseases.

## 1. Background

Infections caused by papillomaviruses (HPVs) are the most common sexually transmitted diseases worldwide. It has been estimated that both men and women are affected by HPV infection at least once in their lives [1]. Also, almost one in three men are infected with at least one genital HPV, and one in five are infected with oncogenic HPV genotypes [2]. Globally, because of these infections, it is estimated that 625,600 women and 69,400 men have HPV-related cancer each year, and more than 340,000 women die of cervical cancer (CC) [2].

In men, HPV infection tends to manifest clinically as anogenital warts, which cause significant morbidity and increase HPV transmission rates. HPV infections are also associated with penile, anal, and oropharyngeal cancers, which are commonly linked to HPV type 16 [3]. HPVs of the alpha genus are the principal cause of anogenital warts, traditionally called condyloma, but can also cause intraepithelial neoplasia that can lead to cancer (2). HPV16 and HPV18 infections are also involved in a subgroup of head and neck tumors, the incidence of which is constantly growing worldwide, according to epidemiological data [4]. The epidemiological classification of anogenital alpha HPVs divides the viruses into high-risk (HR, HPV16, 18, 31, 33, 35, 39, 45, 51, 52, 56, 58, 59, 66, and 68) and low-risk (LR HPV6, 11, 7, 2, 3, 10, 32, 57, and others) HPVs according to their link to cervical cancer cases observed worldwide [5]. Anogenital warts are proliferative and benign lesions found in any part of the genitalia, in mucosal or cutaneous epithelia, the anus or perianal area, and the inguinal or pubic regions. They are 90% caused by LR HPV6 and HPV11 [6], but many other LH-HPV types have been detected in the warts of HIV-positive individuals [7]. HPVs are also responsible for laryngeal papillomatosis (LP), also known as recurrent respiratory papillomatosis (RRP), a rare and chronic disease, the most common benign neoplasm in children characterized by grape-like exophytic papillomatous lesions in the larynx and respiratory tract [8]. Patients undergo multiple surgeries to periodically remove exophytic papillomas and prevent airway obstruction. Most cases caused by HPV6 and 11 genotypes are benign; however, HR genotypes (HPV16, 18, 31, and 33) can be associated with an increased risk of malignant transformation [9,10]. All HPV-related diseases are severe in HIV-positive individuals or with defects in their immune systems, including those receiving immunosuppressant drugs [11,12]. A reduction in the incidence of genital warts has been recorded as an immediate consequence of the introduction and implementation of quadrivalent (HPV 6, 11, 16, and 18) and nonavalent HPV (HPV, 6, 11, 16, 18, 31, 33, 45, 52, and 58) vaccines in several countries [13,14].

Genotypes belonging to all human HPV genera (alpha, beta, gamma, mu, and nu) cause cutaneous warts. They may occur on any part of the body. Skin warts are characterized by a typical morphology, name, location, and specific HPV genotype, although the genotype is not usually diagnosed. HPV2, 4, 7, 26–29, and 57 are detected in common warts on the feet, hands, fingers, and lips; HPV3, 10, 27–29, and 41 are detected in flat or plane warts. These occur grouped from two to hundreds and are localized to the face and dorsum of the hands; mosaic warts (HPV2) are present on the soles and are painless but resistant to treatments; Myrmecia warts (HPV1) are painful palmar and plantar warts; filiform warts (HPV3) are present on orifices, face, in the beard and periungual regions, and are gray, brown, or flesh colored; endophytic warts (HPV4) are present as a group on either palmar or plantar areas, show a central depression, and have a hard keratin mass; and butcher’s warts (HPV7) are large, cauliflower-like warts, which are detected frequently on people who work with meat [15]. (The genus of single HPV can be found here: https://pave.niaid.nih.gov/, accessed on 5 March 2024).

Anogenital and skin warts, although benign, are recurring and recalcitrant lesions. They cause the patients considerable suffering and psychological stress and also represent significant costs for the healthcare systems. Although it has been proven that warts heal in 2 years and that 50% of children affected by skin warts heal in 6 months, warts are infectious lesions that must be treated to avoid their spread in the community causing actual outbreaks [16,17]. Moreover, anogenital warts are the most common sexually transmitted infection, so their treatment is a priority. A wide variety of treatments and guidelines are available to manage cutaneous and genital warts [18,19,20]; however, a percentage of lesions become recalcitrant, probably depending on the host factors (immunocompetence) and viral factors (genotype involved). Recurrence after the surgical treatment of anal condyloma acuminata has been estimated in 25% of cases [18] and in 44.35 of genital warts in unvaccinated men; thus, an antiviral treatment that can lead to the resolution of an HPV infection would be very useful [19].

This report presents clinical cases demonstrating remissions of HPV-related pathologies following treatment with Acyclovir (ACV) or Valacyclovir (VCV) in oral or topical forms based on our experience and literature reports. The paper describes original data regarding the remission of genital warts and cervical and vaginal lesions, including one case of vulvar giant condylomata acuminata of Bushke–Löwenstein (GCBL) [20]; additionally, it covers a literature review of clinical cases and pilot studies on skin warts and laryngeal papillomatosis treated with ACV or VCV, to the best of our knowledge.

ACV and VCV are synthetic purine nucleoside analogues with inhibitory activity, in vitro and in vivo, against viruses of the Herpesviridae family, including human herpesviruses 1 and 2 (HHV-1 and HHV-2) and, to a lesser extent, varicellae zoster virus (HHV-3) [21]. Recently, additional activities have been shown for ACV regarding cellular metabolism. Therefore, old and new ACV mechanisms of action will be discussed to determine their possible effectiveness when treating HPV-dependent lesions.

## 2. Acyclovir Treatment of Condyloma and Giant Condyloma Acuminate of Buschke and Lowenstein Clinical Cases

The clinical efficacy of ACV on anogenital lesions caused by HR and LR-HPV was accidentally observed during antiviral treatments of patients with vaginal or vulvar herpetic blisters. Patients with recurrent genital lesions, subjected to ineffective therapies, received a colposcopy examination and a diagnosis for HPV-related infections along with genital HHV infections. The efficacy recorded in a case series of LH- and HR HPV infections has been recently described [22]. Seven patients with clinical signs of HHV-2 and vulvo and/or vaginal candidiasis were treated with ACV or VCV by oral route administration or by a 5% ACV topical route. Patients showed HPV-related lesions, such as vulvar, perineal, or anal micro-condylomatosis, and the squamous intraepithelial lesion (SIL) of the cervix. The treatment was personalized, the patients received a follow-up session with a colposcopy examination, and the pharmacological treatment was interrupted when the lesions disappeared. The healing time for HPV lesions varies in different patients. It starts from a minimum of 2 weeks in a patient 40 years old with vulvar HPV lesions treated by both oral and topical ACV to a maximum of 7 months in a patient 48 years old at risk of kidney and liver failure with an LSIL and DNA test positive for HPV16 treated only with vaginal 5% ACV [22].

The clinical efficacy of ACV on HPV lesions was also observed in male patients without apparent HHV-2 lesions. They were invited to a control visit to prevent and observe a couple’s treatment of STIs detected in the partners. Table 1 reports the characteristics of the male patients with the lesion type and the antiviral treatment, oral or topical, offered until a *restitutio ad integrum* of the penis without any scars.

During anamnesis, patients ID30 and 36 reported that they had been suffering from condylomas for a long time and had received either cryoscopy or laser treatments. Unfortunately, condyloma relapses after both treatments. The five male patients were treated with oral and topical ACV (ID11 and 30) or only topical ACV (ID36 and 39). The dosage of ACV, present in the D and E columns, was decided according to the health of the patients and its compatibility with drug administration. The treatment time was different among the five patients (F column). It was shorter in patients who received both oral and topical treatments compared to the remaining patients who received only topical treatments. ID 36 and 39 received galenic preparations of ACV at 8% and 7%, respectively, in two different drug vehicles specific for cutaneous tissue. These were Transcutol (Gattefossè, Saint-Priest, France), a highly purified diethylene glycol monoethyl ether associated with skin penetration enhancement in topical dosage forms, and Pentravan^®^ (Fagron, Rotterdam, The Netherlands), which is an emulsion that uses liposomal technology to ensure transdermal drug delivery.

**Figure 1:**
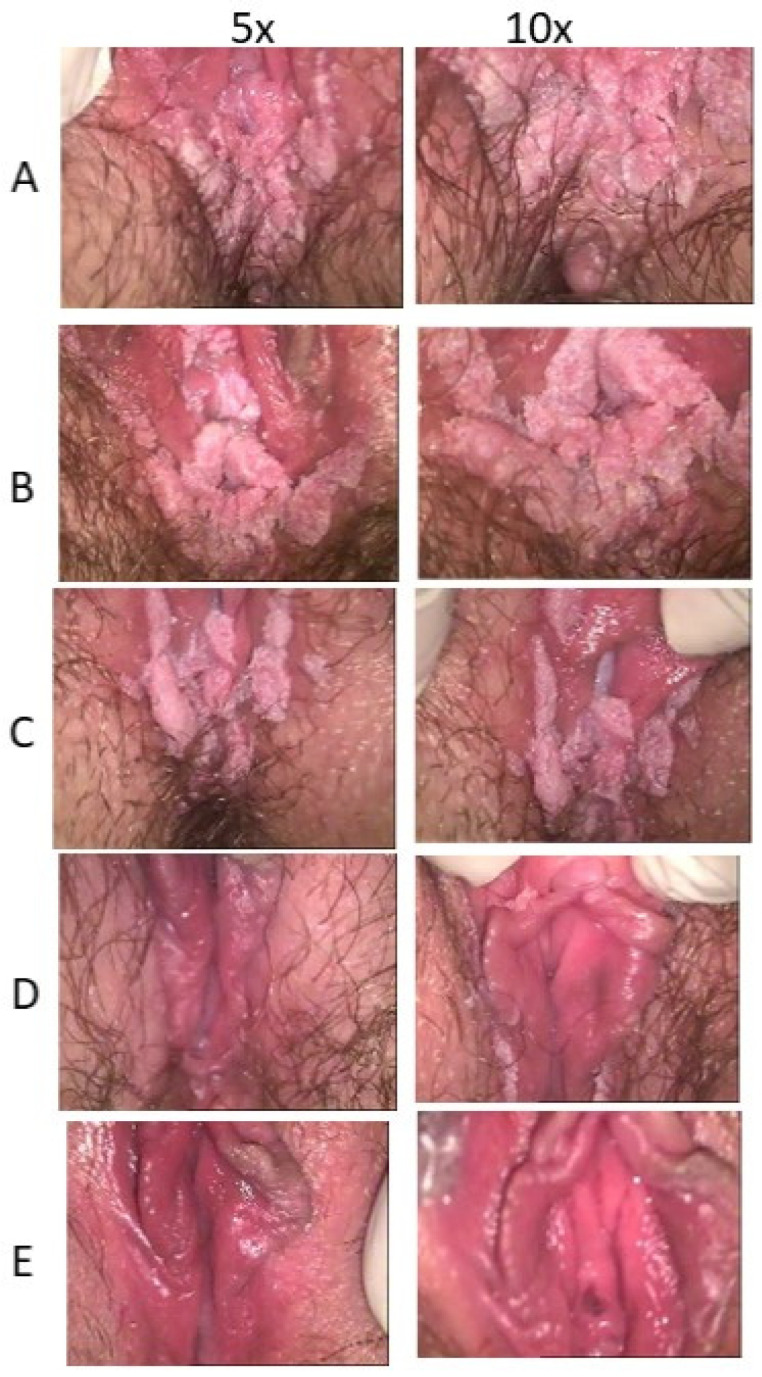
Colposcopy images of the ID5 patient’s vulva at the first visit (**A**); (**B**–**D**) are representative images showing GCBL remission during the ten months of follow-up. Image (**E**) shows the restitutio ad integrum after 1 and 2 years from the first visit.

**Figure 2:**
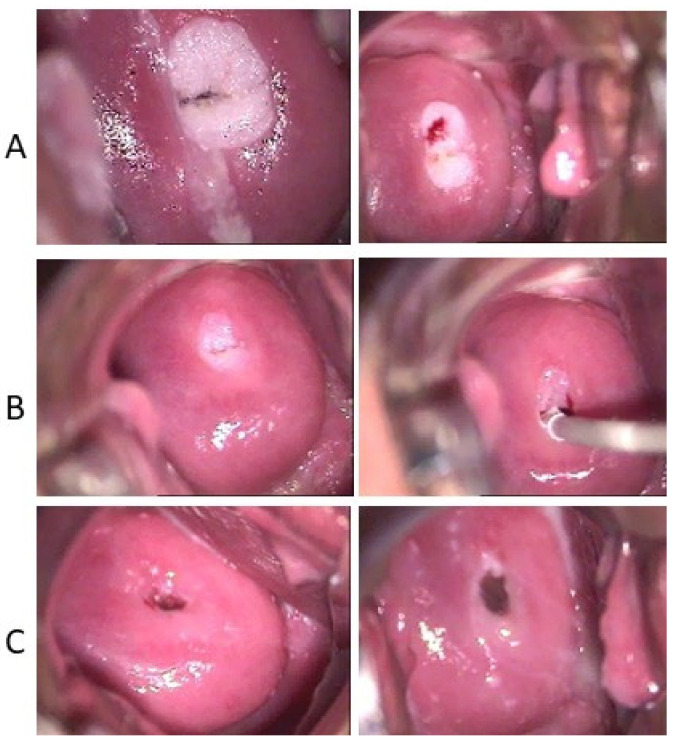
Colposcopy images of ID5′s cervix after application of a 3% acetic acid solution. The histology test confirmed that the white lesion is a condyloma acuminata. Image (**A**): cervix at the first visit (10×); in the right panel, the sign of histological sampling; (**B**) images (5×) show the cervix during the treatment and the follow-up visits. (**C**) images (5×) show the *restitutio ad integrum* of the cervix after ten months after the first visit.

**Figure 3:**
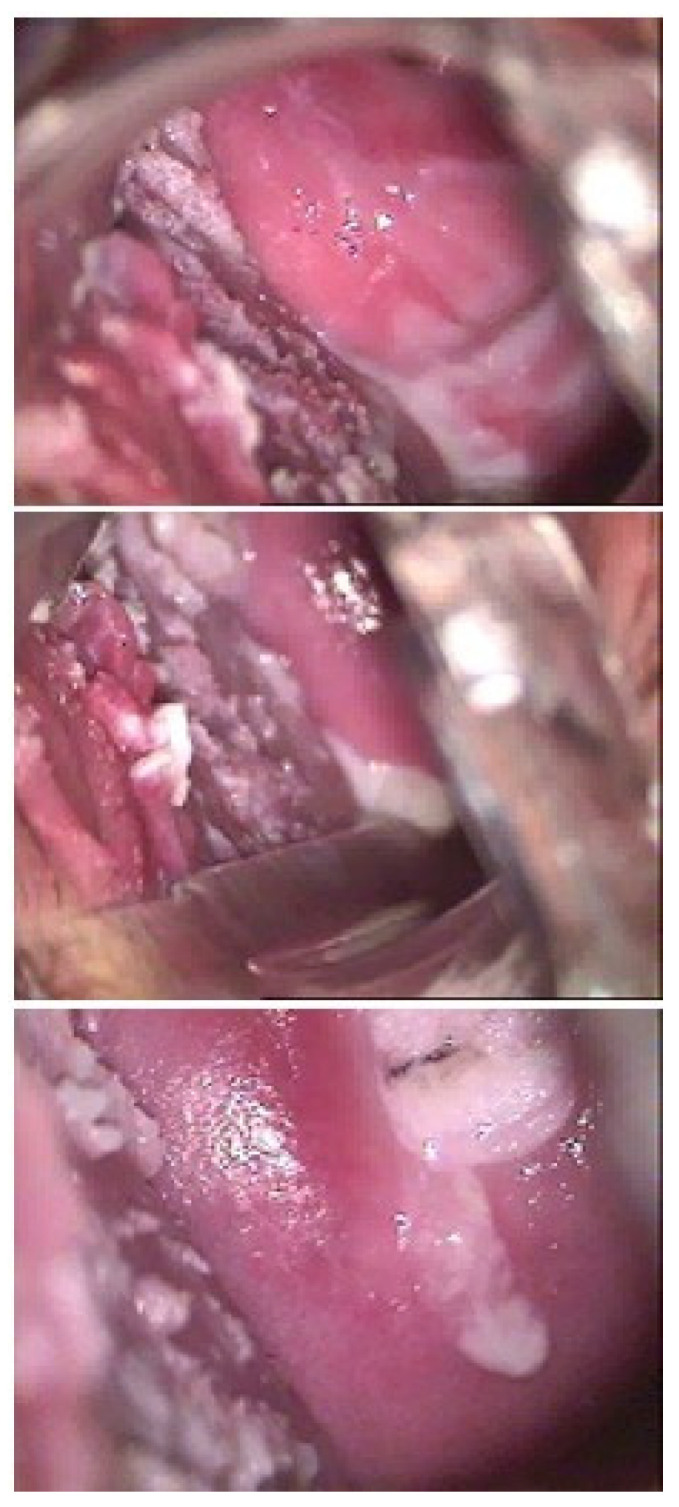
Vaginal condyloma acuminate of ID5.

The clinical efficacy of ACV has also been observed in one young woman with vulvar giant condyloma acuminates of Buschke and Lowenstein (GCBL) without evidence of HHV blisters. The clinical characteristics are in Table 2. The young woman (ID5) was treated with ACV and VCV as a last resort because she received a diagnosis of GCBL and a proposal for surgery of the vulva. At the first visit, the patient showed: (i) vulvar GCBL with a characteristic subversion of the tissue (Figure 1A), (ii) a flat condyloma of the cervix (Low-SIL) confirmed by a histology exam and tested positive for a LR-HPV genotype unknown by the assay used (Figure 2); and (iii) a vaginal condyloma acuminated white for a co-infection with candida (Figure 3). The woman received oral and topical administrations of ACV, VCV and weekly follow-up visits. The first treatment consisted of the administration of 2.4 g/day ACV for 30 days. The second treatment consisted of 2 g/day VCV for 40 days. Week after week, it was possible to observe in ID5 a remission of both vulvar and cervical lesions until *restitutio ad integrum*. Figure 1 shows colposcopy images of the vulva, which shows the progressive remission of GCBL. Figure 2 shows colposcopy images of the cervix of the same patient, showing progressive remission from condyloma acuminate. Image B shows the cervix during drug treatment, showing the remission of the lesion and complete resolution. No recurrence was observed in the following two years and over for any HPV-related lesions.

## 3. Acyclovir Treatment of Cutaneous Warts (Verrucas)

Several anecdotal cases of ACV treatment of warts have been described since the early 1980s. The first description was the case of a young woman with sporadic episodes of psoriasis, affected by recurrent plantar warts for seven years, starting from the age of 11 years old. She was cured without success with (1) weekly applications of salicylic acid followed by destruction by cauterization, (2) chirurgic excision and cauterization under local anesthesia, (3) treatments with salicylic acid paste followed by solid carbon dioxide, and (4) formalin soakings for two months. Unfortunately, the wart continued to spread, rising 5 mm above the skin’s surface and making walking painful. As a last resort, ACV treatment was suggested, and the young woman was treated with 2.5% ACV ointment under cellophane occlusion applied to the affected area every two days. After one week, the lesion was no longer painful; after two weeks, the wart’s surface became irregular, and after eight weeks of treatment, pieces of the wart became detached. It is unclear how long the treatment was beyond eight weeks, but the complete resolution of the wart lesion was obtained without any more recurrences during the follow-up period [23]. One year later, three cases of successful treatment of warts with ACV were described [24]. Two men and one woman were referred to the clinician to treat mosaic plantar warts recalcitrant to monthly topical acid therapy (mono- and bi-chloroacetic acids). Warts covered 10–20% of the plantar surface and were treated every morning with an aluminum hexahydrate solution (Xerac-AC) and ACV 5% ointment (Zovirax) three times daily for seven- to ten-day intervals. Two patients were free of warts after three sessions, and the third patient after the fourth section. There was no recurrence in the next 4–6 months of follow-up observations. Two years later, Hurwitz RM described a successful treatment with ACV ointment of recalcitrant warts in 24 individuals. The clinician used ACV ointment to treat a variety of warts located on the hands, feet, and genitals in children and adults of different ages. Within eight weeks of applying ACV, 38% of the patients were completely cleared of warts, while 59% showed a 90% decrease in lesions [25]. All the authors of the abovementioned papers concluded that, although warts can heal without any treatment or placebo effect, ACV has proven effective in treating recalcitrant and recurrent warts, suggesting the need for double-blind controlled trials.

In the same year, a randomized, double-blind study was performed on 52 subjects with a clinical diagnosis of verruca plantaris. The study compared the effect of ACV cream (Zovirax) versus a placebo cream and liquid nitrogen. The ACV dose was 5% in a modified aqueous cream, administered once daily for six weeks. After eight weeks of treatment, 7 of 18 (ACV cream), 5 of 18 (placebo cream), and 1 of 11 (liquid nitrogen) patients were cleared of their warts. The authors concluded that ACV is not better than a placebo for treating warts and that liquid nitrogen should not be considered for routine treatment [26].

Nevertheless, another paper about ACV efficacy on plantar warts by topical administration was published after a few years. A father and son affected by diffuse verruca planas on the face (9-year-old child) and on the hand (55-year-old man) were treated with topical applications of ACV every three hours. After 30 days of treatment, the number of warts decreased in both individuals, and after 60 days, the warts disappeared completely. No relapse was observed in the 2-year follow-up session [27]. In the 2000s, two different papers reported accidental observations of warts healing in three individuals after treatments with ACV [28] and VCV [29] administered by the oral route in herpesvirus (HHV-2 and HHV-3) cases. A 30-year-old man affected by genital herpesvirus (HHV-2) was treated during the recurrences with VCV 1 g/die for a week. The patient had plantar warts for three years. During his last HHV2 recurrence, he noticed significantly improved warts after using VCV. He continued the treatment for 37 days until the warts disappeared. In a second case, an adolescent male suffered from painful plantar warts for four years. He was treated without success for three months with a topical salicylic acid/lactic acid combination by cryotherapy and for four months with topical imiquimod 5% cream BID under an occlusive bandage before being treated with oral VCV. The lesions disappeared after two months of treatment with 1 g/die VCV [29]. The last case regards a 49-year-old woman affected by recurrent plantar warts. As they were painful, they were treated six times with surgical debridement followed by monochloroacetic acid for seven months. The woman was then diagnosed with Herpes zoster (HHV-3) and treated for ten days with 4 g/die of ACV. After this treatment, the podiatric warts gradually disappeared [28].

More recently, intralesional injection of ACV has emerged as an option for warts treatment. In a non-randomized controlled study, 31 patients affected by finger, hand, plantar, or periungual warts were treated with ACV. Half of the patients received previous treatments with cryotherapy, trichloroacetic acid (TCA), and laser therapy. A group of 19 patients was treated with injections of ACV (7 mg into the base of each wart) every two weeks for a maximum of five treatments. In contrast, the control group of 12 patients received intralesional injections of saline solution. The result shows a statistically significant difference between the treated and the control groups. In the ACV group, a 100% clearance of warts was obtained for 52.6% of the individuals, a partial clearance for 36.8%, and no effect for 10%. In the control group, only one person of 12 had their warts cleared. A possible different effect of ACV on hand warts versus plantar warts was observed. The clearance rate with ACV was comparable to that obtained with 5-fluorouracil (65%) and bleomycin (58%), and it was lower than cidofovir (90% to 98.5%), a new, expensive antiviral treatment and not available in the authors’ country [30]. Another study was performed comparing the efficacy of the intralesional administration of ACV (Acivir, Cipla) versus intralesional tuberculin PPD (ARKRAY), an adjuvant peptide partially effective for the treatment of warts [31]. Patients were affected by common, palmar, plantar, periungual, and subungual warts. Twenty of them (group A) were treated with 0.1 mL of tuberculin PPD (units not indicated) every two weeks for a maximum of six sessions (12 weeks), and 20 patients received intralesional injections of ACV (7 mg/wart) for the same time. Warts treated with either intralesional ACV or PPD showed a complete resolution in 60% and 30% of individuals, a partial resolution in 25% and 30%, and no effect on 15 and 35% of individuals. These differences between the groups were not significant (*p*-value > 0.05). However, the authors noticed a different response of ACV on various types of warts. They suggested intralesional ACV as a better option for treating periungual and subungual warts [32].

## 4. Acyclovir Treatment of Laryngeal Papillomatosis

The first report describing a successful postoperative treatment with ACV of laryngeal papillomatosis (LP) [33], also known as recurrent respiratory papillomatosis (RRP), was published in 1991 regarding the treatment of 5-, 7-, and 11-year-old children. The first child showed dysphonia and dyspnea, mainly at night, for two months. A mass in the glottis close to the vocal cords was diagnosed as LP and removed using forceps. After three months, micro-laryngoscopy confirmed the extent of the tumor, and a total excision was again performed using forceps. After surgery, the child underwent treatment with ACV 300 mg/day by intravenous injection for five days, and no recurrence of LP was detected. The patient had a normal laryngeal function in the 42 months of follow-up. The second child, affected by dysphonia and an unproductive cough for 12 months, presented an LP in his vocal cords. The mass was completely excised using forceps, and the patient underwent treatment by oral route with ACV 600 mg/day for five months. No recurrence was observed during the 18 months of the follow-up period. A third child showed dysphonia and dry cough for 15 months and presented a papillomatosis mass in the glottis and vocal cord areas. The mass was entirely excised with forceps. The patient was postoperatively treated with ACV 500 mg/day orally for 30 months; no side effect or recurrence of LP was observed in this period [33]. In a second study, 12 patients, seven females and five males, with ages ranging from 3 to 25 years old (mean age: 12 years old) affected with an aggressive RRP, were orally treated with ACV the day after endoscopic microsurgery with CO_2_ to completely remove the exophytic form of papilloma. The dose administered was 400 mg/day for patients under five years old and 800 mg/day for patients aged 6–25 years old. The patients in this study had undergone up to 15 surgeries in the past. After receiving ACV treatment, they remained disease-free for a significantly more extended period than the expected interval between their previous surgeries [34]. A pilot study on ACV administration was performed on patients with severe, recalcitrant, juvenile-onset recurrent respiratory papillomatosis treated regularly with laser ablation therapy. Patients were treated with the antiviral for 52 days. The dose for ACV was 10 mg/kg, five times a day for ten days, followed by twice daily for 42 days. The study showed a statistically favorable response of the patients to ACV administration, except for two patients who switched from interferon to ACV and experimented with a rebound phenomenon with the worsening of diseases [35]. Chaturvedi J and co-authors reported three severe cases of RRP in adults treated with ACV along with oral prednisolone. After micro-laryngeal surgery with the excision of papillomatous lesions using a laryngeal microdebrider, the patients were treated with oral ACV 800 mg five times/day for five days along with oral prednisolone at 1 mg/kg body weight for three days. This protocol was repeated each month for one year. Patients showed a significant improvement after the surgery and did not experience any papillomatosis recurrence for up to a year. Additionally, no adverse effects of the medication were reported. The study found that administering antiviral drugs at regular intervals, with a short course of oral steroids, can result in rapid recovery and prevent the activation of latent viruses within the laryngotracheal system, thus ensuring long-term improvement [36]. In another study, 21 children with juvenile-onset RPP underwent a primary surgical excision of the papilloma, followed by oral ACV treatment. The patients received oral ACV for two months at a dose of 80 mg/kg/d in divided doses (max: 3200 mg/d). It was observed that using ACV as a postoperative adjuvant significantly reduced the mean interval between surgeries and the number of surgical interventions required compared to surgery alone. The authors concluded that postoperative systemic ACV can prolong disease control and avoid repeated surgeries [37].

## 5. Acyclovir and Valaciclovir: Mechanism of Action

Antiviral ACV (9-[(2-hydroxyethoxy)-methyl]-guanine) was discovered in 1974 and patented in 1977 by Burroughs Wellcome. After successful clinical trials, the drug became available to physicians in 1981. VCV was patented in 1987 by the same company and came into medical use in 1995 when GlaxoSmithKline marketed it [38]. ACV and VCV have been included in the list of Essential Medicines by the World Health Organization (WHO/MHP/HPS/EML/2023.02) and are now both generic drugs. VCV is an L-valyl ester of ACV converted to ACV after oral administration by intestinal and hepatic metabolism. It has a 3–5-fold higher plasma level than ACV, resulting in higher bioavailability and less frequent dosing. The drugs are active against HHV-1, HHV-2, and, to a lesser extent, HHV-3 (Varicella zoster virus). The drugs also show minor activity against the Epstein–Barr virus (HHV4) and Cytomegalovirus (HHV-5). The measurement of ACV’s inhibition constants (*Ki*) derives from biochemical studies in the 1980s, with DNA polymerase enzymes purified, or only partially purified, from HHV-1/2-infected cells. Viral enzyme activity was compared with the activities of cellular polymerase alpha (pol α) and beta (pol β) purified from uninfected cells of various origins. Biochemical studies have been conducted in different labs over the years to measure the *Ki* (inhibition constant) of ACV. There have also been discrepancies between the *Ki* measurements obtained by these studies. However, it was clearly shown that ACV is a potent viral polymerase inhibitor compared to the inhibition obtained with cellular pol α and pol β. The variability in the *K_i_* values reported for cellular enzymes may be attributed to differences in the experimental design, viral strain, and cell source used for enzyme isolation [38]. The antiviral activity was never re-evaluated, despite the possibility of producing recombinant proteins for better standardizing enzymatic assays. To summarize, ACV is phosphorylated in infected cells by a herpetic thymidine kinase. ACV monophosphate is then further phosphorylated by cellular enzymes in the infected host, forming ACV diphosphate and triphosphate forms. The latter form selectively inhibits the activity of the viral DNA polymerase, preventing viral DNA replication and probably also host DNA synthesis to a lesser extent. Whether the first ACV phosphorylation can occur by cellular enzymes, such as TK1 [39], without HHV infections has to be demonstrated. Dr Gertrude B. Elion, who discovered ACV, reported that, in phosphorylation studies using labeled-ACV in cell cultures, a small level of ACV-mono-phosphorylation was measured due to cytosolic 5′-Nucleotidase (EC 3.1.3.5), that exchange –P– group among nucleotides [40], an activity probably attributable to the soluble form of human ecto-5′-nucleotidase, without a GPI anchor, also known as NT5E (CD73). However other 5′-Nucleotidase isoforms might be involved.

Recent studies have revealed additional functions for ACV as an adjuvant in cancer chemotherapy [41]. ACV affects growth kinetics, cell proliferation indices, cell survival, and micronuclei induction in cervical and breast cell lines [42,43]. ACV exhibits antitumor activity targeting βTrCP1, one of the four subunits of the ubiquitin protein ligase complex. It has been proposed that ACV abolishes βTrCP1 ligase activity toward the ubiquitination of substrates [44]. Interestingly, is upregulated by the HPV16 E7 oncoprotein [45] and interacts not only with E7 from high-risk alpha HPVs (HPV16, 18, 33, and 39), but also with E7 from beta HPV genotypes (HPV8, 38, and 49), indicating that this target of the HPV protein may be necessary for viral persistence and carcinogenesis [46].

**Figure 4:**
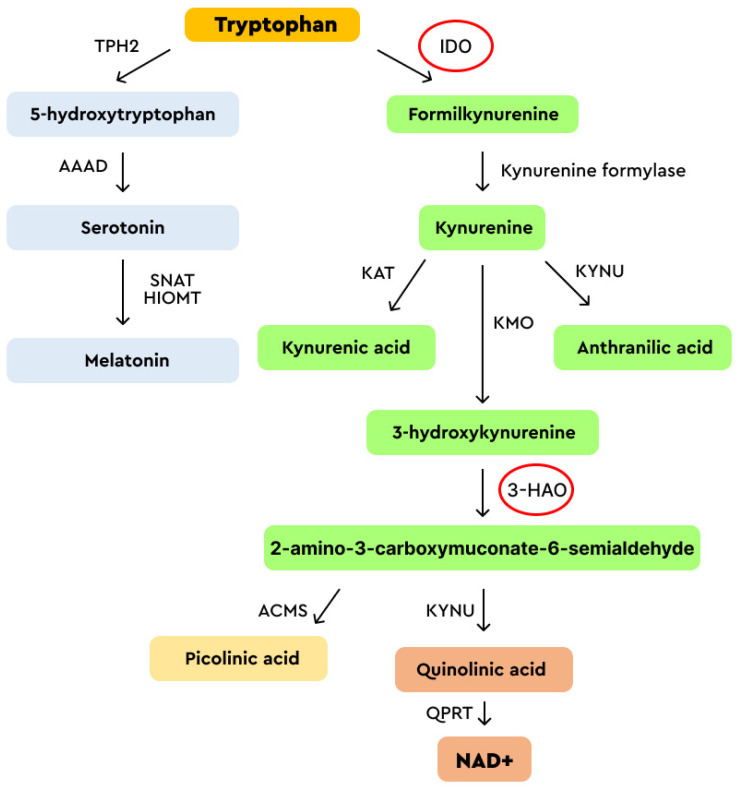
Tryptophan (L-Trp) metabolism in mammalian cells. The enzymes affected by ACV are circled in red. IDO: indoleamine 2,3-dioxygenase; KAT: kynurenine aminotransferase; KYNU: L-kynurenine hydrolase; KMO: kynurenine 3-monooxygenase; 3-HAO: 3-hydroxyanthranilate-3,4-dioxygenas; ACMS: aminocarboxymuconate-semialdehyde decarboxylase; QPRT: quinolinate phosphoribosyl transferase; TPH2: tryptophan hydroxylase 2; AAAD: aromatic L-amino acid decarboxylase: SNAT: serotonin N-acetyltransferase; HIOMT: hydroxy indole-O-methyltransferase.

By investigating the neurologic protective effect of ACV in a rat model of HHV-1 infection, it has been shown that ACV significantly reduces Fe^2+^-induced lipid peroxidation and O^−2^ generation. ACV reduces O^−2^ generation in the presence of quinolinic acid [45]. ACV binds strongly with Fe^2+^ and Fe^3+^ ions, almost as well as EDTA. As a consequence, ACV reduces the activity of two enzymes, indoleamine 2,3-dioxygenase (IDO) and 3-hydroxyanthranilate-3,4-dioxygenase (3-HAO) [47], both involved in tryptophan (L-Trp) metabolism. IDO antiviral activity has been observed in CMV infection, where its activation is responsible for the inhibition of the growth of CMV in retinal pigment epithelial cells [48]. Moreover, the murine model suggests the relevant involvement of IDO in HPV immune evasion [49]. In patients affected by condyloma acuminata, a high IDO expression abnormally localized in the lesions might be involved in forming a local immunotolerant microenvironment [50]. Tryptophan concentration is perturbed in many infectious and cancer diseases [47,51]. In mammalian cells, L-Trp is metabolized via the kynurenine (Kyn) and serotonin pathways (Figure 4). The Trp/Kyn balance appears modulated in many physiological states and diseases, and it is emerging as a universal biomarker for health [52].

The IDO enzyme is involved in several activities and is found to be dysregulated in HPV-related diseases [50,53,54,55,56]. 3-HAO is a non-heme iron-dependent enzyme that catalyzes quinolinic acid synthesis, a downstream product of the Kyn pathway [57]. Quinolinic acid is converted by quinolinate phosphoribosyl transferase (QPRT) to NAD+, a coenzyme for enzymes involved in energetic metabolism, DNA repair, gene expression, and stress response [58].

In addition, ACV has been shown to form metal complexes with many other divalent ions, such as cobalt, nickel, copper, zinc, cadmium, and platinum [59,60,61], known cofactors of many cellular enzymes. ACV maintains antiviral activity, even when it forms complexes with divalent ions [62].

## 6. Discussion and Conclusions

A case series involving individuals affected by HPV-related diseases who benefited from using antiviral ACV has been described. Antiviral ACV was administered orally and/or topically. Most of the HPV-related conditions were caused by LR-HPV. Several individuals had recurrent lesions that showed remission by using the antivirals. The primary objection to the data presented is that condyloma and LR-HPV lesions may naturally progress into remission spontaneously. However, we closely monitored our patients weekly and observed clear signs of recovery within a few weeks of antiviral treatment. These signs included the elimination of itching in the lesions, a reduction in their redness, and a decrease in the size of the condylomas.

To support our clinical findings, we conducted a literature review and included several case series and small pilot studies. ACV and VCV have been used to treat two main HPV pathologies, skin warts and laryngeal warts, since drug commercialization. All authors of the considered studies concluded that randomized clinical trials were necessary to determine the efficacy of ACV. However, no study has been completed yet. After analyzing the clinical cases reported, differences have emerged in the dosing and the antiviral administration time. The studies on using ACV as postoperative adjuvant therapy in RRP are promising. With the topical use of ACV for skin wart treatment, a different result was obtained with the ACV ointment [24,25,26] compared to ACV cream [26]. The choice of a vehicle to achieve a good penetration of the drug into the skin may be a critical point, and the right vehicle will need to be evaluated to demonstrate ACV’s efficacy on HPV-related diseases; a different drug vehicle may be required to penetrate various kinds of warts compared to HHV-1/2 blisters [63]. The morphological characteristics of warts and the HPV-related genotype could influence treatment outcomes and drug efficacy, as suggested in [64]. Finally, the intralesional injection of ACV has emerged as the best delivery method for treating warts. Recently, three clinical trials on the intralesional use of ACV for plantar wart therapy are available at https://clinicaltrials.gov/ (accessed on 14 March 2024). Three Egyptian public universities, namely Assiut University, Cairo University, and Sohag University, are sponsors and responsible for conducting these trials. The first study (NCT05324904) compares the effectiveness of intralesional vitamin D3 administration with intralesional ACV. The second study (NCT06261684) is a randomized trial that compares the efficacy of intralesional ACV with cryotherapy treatment, and the third study (NCT05429151) compares the effectiveness of the intralesional injection of ACV with Candida Antigen treatment for plantar warts. The interest of clinicians in the use of ACV and VCV to treat HPV-related diseases resides in the fact that these antivirals are generic drugs, not more patented, and are available at a lower cost compared to newer antivirals, like cidofovir, which is 100-times more expensive and not available in many low-income countries [30,32].

Beyond the canonical mechanism of the antiviral action of ACV in HHV-1-2-infected cells [40], additional effects have been shown for ACV on cellular metabolism, which might be implied in its effectiveness toward HPV-dependent lesions. ACV can affect cellular metabolism by chelating divalent ions such as iron, cobalt, nickel, copper, zinc, cadmium, and platinum [59,60,61]. These ions are essential cofactors for metabolic enzymes. In this context, ACV reduces the activities of IDO and 3-HAO enzymes in tryptophan metabolism. L-Trp metabolism produces several essential molecules for host physiology that are perturbed in neurological, psychiatric, metabolic, infectious, intestinal, and cancer diseases, making it an ideal pharmacological target [49,51].

ACV exhibits antitumor activity targeting βTrCP1, one of the four ubiquitin–protein ligase complex subunits, which regulates multiple cellular processes mediating the degradation of various targets [44,45]. E7 of several HPV genotypes targets βTrCP1, modulating the proteasome activity of the infected cells, suggesting a possible effect of ACV on the HPV life cycle.

## Figures and Tables

**Table 1:** Clinical characteristics of the patients treated for condylomatosis. Columns from left to right report: A: identification number (ID); B: age; C: HPV lesion type; D: oral dosage of ACV; E: topical treatment of ACV; colposcopy images. ACV: acyclovir; d: day; m: month.

ID	Age	HPV Lesion Type	Oral ACV Dosage	Topical ACV	Treatment Time (d,m)	Colposcopy Image
A	B	C	D	E	F	G
11	30	Frenulum penile condylomatosis	ACV 1600 mg/20 d	5% ACV (20 d)	20 d	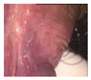
30	32	Penile condylomatosis (recurrence at cryoscopic treatment)	ACV 1600 mg/20 d	5% ACV (2 × 30 d)	2 m	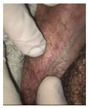
36	41	Penile condylomatosis (recurrence at laser treatment)	_	8% ACV Transcutol (2 × 45 d)	4 m	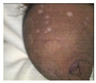
39	29	Penile micro-condylomatosis	_	7% ACV Pentravan (2 × 45 d)	3 m	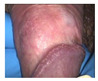
41	37	Penile micro-condylomatosis	_	8% ACV Transcutol (2 × 45 d)	1 m	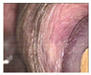

**Table 2:** Clinical characteristics of the patient and ACV treatment.

ID	Age	Cervical Lesions	HPV Lesion	Oral Dosage (mg/d)	Topical Dosage	Treatment Time (Month)
	A	B	C	G	H	I
5	27	Condyloma acuminate LR-HPV	Vulvar GCBL	ACV 2.4 g/30 d, VCV 2 g mg/40 d	5% ACV (2 × 60 d)	5

## Data Availability

Clinical data can be viewed by writing to M.B.
